# Reprogrammed Transcriptome in Rhesus-Bovine Interspecies Somatic Cell Nuclear Transfer Embryos

**DOI:** 10.1371/journal.pone.0022197

**Published:** 2011-07-25

**Authors:** Kai Wang, Hasan H. Otu, Ying Chen, Young Lee, Keith Latham, Jose B. Cibelli

**Affiliations:** 1 Michigan State University, East Lansing, Michigan, United States of America; 2 BIDMC Genomics Center, Harvard Medical School, Boston, Massachusetts, United States of America; 3 Department of Bioengineering, Istanbul Bilgi University, Istanbul, Turkey; 4 Temple University, Philadelphia, Pennsylvania, United States of America; 5 Programa Andaluz de Terapia Celular, Andalucia, Spain; Wellcome Trust Centre for Stem Cell Research, United Kingdom

## Abstract

**Background:**

Global activation of the embryonic genome (EGA), one of the most critical steps in early mammalian embryo development, is recognized as the time when interspecies somatic cell nuclear transfer (iSCNT) embryos fail to thrive.

**Methodology/Principal Findings:**

In this study, we analyzed the EGA-related transcriptome of rhesus-bovine iSCNT 8- to 16-cell embryos and dissected the reprogramming process in terms of embryonic gene activation, somatic gene silencing, and maternal RNA degradation. Compared with fibroblast donor cells, two thousand and seven genes were activated in iSCNT embryos, one quarter of them reaching expression levels comparable to those found in *in vitro* fertilized (IVF) rhesus embryos. This suggested that EGA in iSCNT embryos had partially recapitulated rhesus embryonic development. Eight hundred and sixty somatic genes were not silenced properly and continued to be expressed in iSCNT embryos, which indicated incomplete nuclear reprogramming. We compared maternal RNA degradation in bovine oocytes between bovine-bovine SCNT and iSCNT embryos. While maternal RNA degradation occurred in both SCNT and iSCNT embryos, we saw more limited overall degradation of maternal RNA in iSCNT embryos than in SCNT embryos. Several important maternal RNAs, like GPF9, were not properly processed in SCNT embryos.

**Conclusions/Significance:**

Our data suggested that iSCNT embryos are capable of triggering EGA, while a portion of somatic cell-associated genes maintain their expression. Maternal RNA degradation seems to be impaired in iSCNT embryos. Further understanding of the biological roles of these genes, networks, and pathways revealed by iSCNT may expand our knowledge about cell reprogramming, pluripotency, and differentiation.

## Introduction

In mammals, the maternal RNA and the proteins present in the oocyte's cytosol are responsible for early embryonic development. These maternal components govern the first embryo cleavages and as they drop by degradation or usage, the zygote nuclei start transcription and protein translation, taking control of embryonic development. This process is called embryonic genome activation (EGA). For mice, bovines and humans, major EGA takes place at the 2-cell, 8-cell and 4- to 8-cell stage, respectively [1]. EGA marks the onset of major developmental events that include embryo polarization, inner cell mass (ICM), and trophectoderm differentiation [2]. Compared to the development of a fertilized preimplantation embryo, a somatic cell nuclear transfer (SCNT)-derived embryo has the added challenge of silencing its somatic-specific genes while reactivating all the embryo-related genes. In doing so, it must also shed its differentiated phenotype and gain a new pluripotent state. These processes take place in approximately the same window of time during which a normally fertilized embryo does it, and relying solely on the maternal proteins and RNAs present in the egg's cytosol. Interspecies SCNT (iSCNT), defined as the procedure by which somatic nuclei introduced into the oocyte's cytosol of a different species, presents a larger biological challenge. Many researchers have reported developing iSCNT preimplantation embryos to different degrees; some of them have achieved significant success [3], [4], which suggests a common reprogramming process across species, at least in mammals.

The rhesus monkey (*Macaca mulatta*), a primate closely related to humans, is often used as a valuable experimental model of human development and diseases. Furthermore, researchers have used it successfully in iSCNT studies [5]. In that research, bovine oocyte cytosol supported rhesus somatic cell reprogramming and the development of preimplantation embryos. Our research objective is to study global transcriptome reprogramming in rhesus-bovine (R/B) SCNTs in the context of EGA and to determine the differences and similarities between same-species SCNT and iSCNT at the molecular level. We hypothesize that by looking at embryos in which reprogramming has taken place successfully — either by fertilization or SCNT — patterns of gene expression failures in iSCNT will emerge, potentially indicating ways to improve the reprogramming procedures in somatic cells.

## Results

### Development of rhesus/bovine (R/B) iSCNT embryo and transcriptome analysis using Affymetrix gene chips

We fused enucleated bovine oocytes with rhesus fibroblasts. Out of 352 fused R/B SCNT embryos in five replicate experiments, 264 (75%) developed to the 8- to 16-cell stage. We used 8- to 16-cell stage R/B iSCNT embryos for gene array analysis.

This study used the Affymetrix gene chip rhesus genome array and bovine genome array. The rhesus array comprised over 52,000 probe sets representing over 47,000 *M. mulatta* transcripts; the bovine array comprised over 24,000 probe sets corresponding to approximately 23,000 bovine transcripts. We used a PCR-based amplification system [6] to amplify cDNA samples from R/B iSCNT 8- to 16-cell embryos, bovine oocytes, rhesus fibroblast cells, and hybridized RNAs in the rhesus genome array. We also hybridized bovine oocytes, bovine SCNT 8- to 16-cell embryos, and iSCNT 8- to 16-cell embryo RNAs in the bovine genome array. Three biological replicates were performed in each sample; the average correlation coefficient between biological replicate arrays was over 0.96, demonstrating high reproducibility (Table S1). Hierarchical clustering of all the samples using complete transcriptional profiling used in the two different chip platforms demonstrated the successful separation of different groups of biological samples (Figure S1).

### Reprogrammed transcriptome in iSCNT 8- to 16-cell embryos

Maternal RNA still exists in bovine 8- to 16-cell stage embryos, despite a dramatic drop in the total amount [7]. To prevent the interference between the newly transcribed rhesus RNA and any residual maternal RNA from bovine oocytes, we subtracted the transcriptome of the bovine oocyte from the transcriptome of the iSCNT. We identified 3,438 up-regulated transcripts in the iSCNT embryo compared to bovine oocyte. Oct4 and Nanog were included in this list, and the FCs were 3.3 and 6.6 folds (LCBs were1.3 and 3.5), respectively; however, Sox2 was not on the list. To further refine the group of 3,438 genes, we subtracted gene expression of a rhesus fibroblast used for iSCNT, which left 2,007 uniquely upregulated genes in the iSCNT embryos, which we called the “reprogrammed iSCNT transcriptome.” Table S2 shows the complete list of 2,007 genes. We used the Ingenuity Pathways Analysis (IPA) system to functionally identify the reprogrammed iSCNT transcriptome. Out of the 2,007 genes, 1704 genes were identified as focus genes in the IPA analysis.

**Figure 1:**
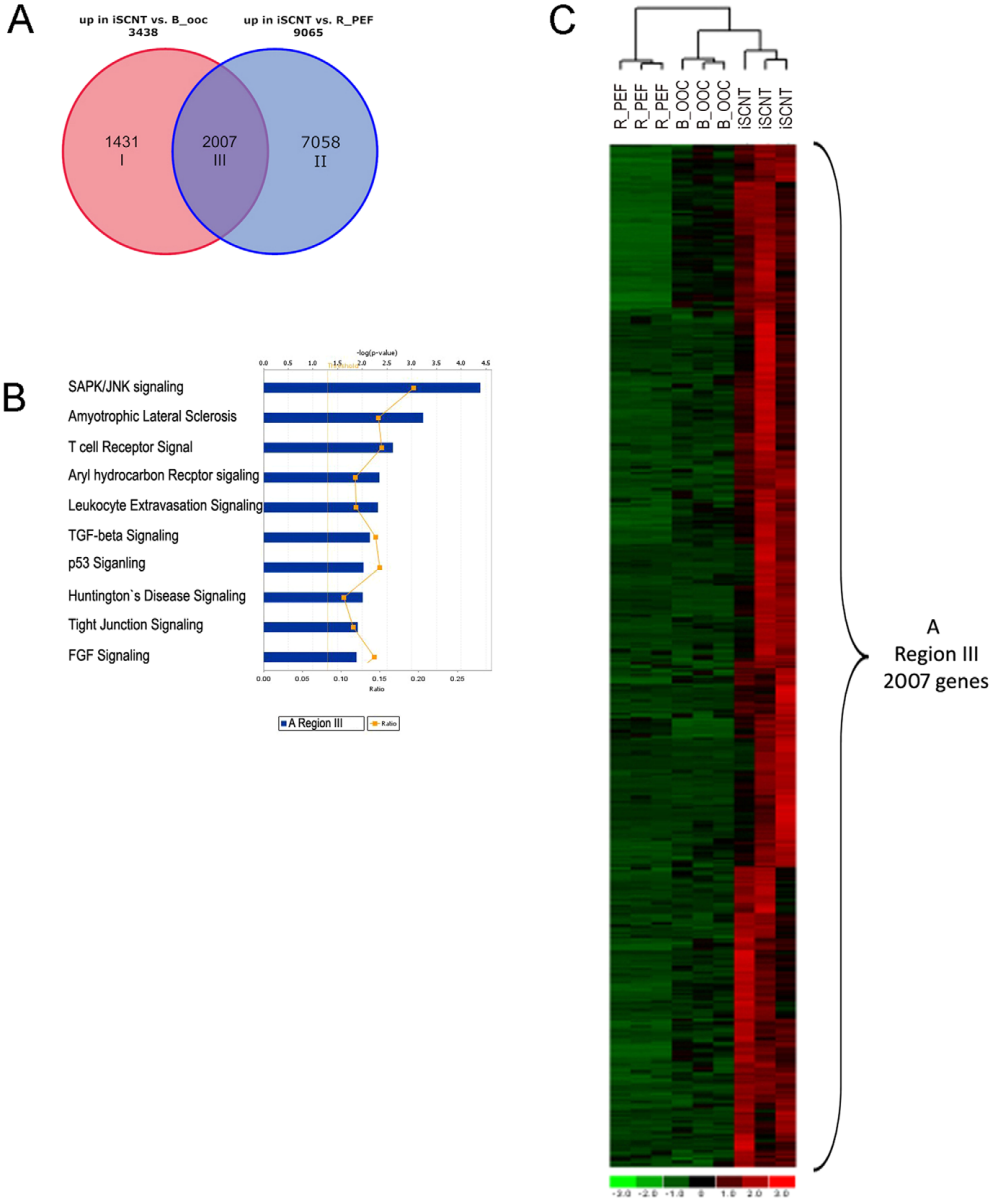
The “reprogrammed transcriptome in R/B iSCNT 8- to 16-cell embryo.” **A.** Venn diagrams showing the “reprogrammed transcriptome in R/B iSCNT 8- to 16-cell embryo” in region III (2,007 genes). The red circle (3,438 genes) indicates the number of upregulated genes in the iSCNT embryo vs. the bovine oocyte (B_OOC); blue circle (9,065 genes) indicates the number of upregulated genes in the iSCNT embryo vs. the fibroblast (R_PEF). **B.** Top ten canonical pathways identified in the reprogrammed transcriptome of iSCNT embryos (Fig. 1A region III, 2,007 genes). **C.** Clustering analysis of samples and genes using reprogrammed transcriptome of iSCNT (Fig. 1A region III, 2,007 genes). Red denotes high and green denotes low gene expression levels.

Tables 1 and 2 and Fig. 1B show the gene network, biofunction, and pathway analyses of the reprogrammed iSCNT transcriptome. The IPA system collects information about how many transcripts are upregulated in each functional category and by how many fold they are regulated; it then calculates the P value and IPA score — the −(log (P value)) — of each function category to describe the level of significance. We identified 51 overrepresented gene networks in the reprogrammed iSCNT transcriptome, using as a threshold a score of 3 or higher and containing 12 or more genes. Table 1 shows the top ten networks with IPA scores higher than 30. Table 2 summarizes biological functions based on identified networks as assessed by IPA. The top functional categories are “gene expression” and “cellular growth and proliferation.” This directly unveils the physiological process of EGA, which happens in the 8- to 16-cell iSCNT embryos. The rest of the eight functional groups mostly relate to “tissue and organism and embryonic development.” The 62 upregulated functional genes groups included POU2F2, Sox9, SIR4, MARK8, MBD3, PAX6 and GATA4— all of which are known to play important roles in preimplantation embryo development. IPA analysis also showed several interesting canonical pathways that may be involved in the reprogramming process of the rhesus donor genome in iSCNT (Fig. 1B). The top ten pathways (Fig. 1B) included SAPK/JNK signaling and TGF-beta, which are well known to be required for embryo development (Kocabas et al., 2006).

**Table 1:** Top 10 networks generated using IPA for the “reprogrammed transcriptome in rhesus/bovine iSCNT (R/B iSCNT) 8- to 16- cell embryos.”

ID	IPA Score	No. of Focus Molecules	Molecules in Top Network	Top Functions
1	43	33	ABTB2, ADNP, C22ORF28, CCR6, CD6, CX3CR1, EEA1, ELAVL2, GDI2, GM2A, GNL1, GPD2, ICEBERG, ICOSLG, IER2, KPNA3, M6PRBP1, MUC5AC, NR6A1, NUP50, OGN, PLP1, PRKAA, Rab5, RAB14, RAB5C, SCUBE1, SEPP1, STAP2, SUPT4H1, TNF, TNFRSF21, TUB, WTAP, ZBTB11	Cellular Development, Cellular Growth and Proliferation, Hematological System Development and Function
2	41	32	AKT2, Alcohol group acceptor phosphotransferase, BAT5, CCR5, CDK6, CSK, Cytochrome c, FOXC1, FZR1, G3BP1, GMNN, GRK6, HIPK3, HM13, HOXA7, HOXB6 (includes EG:3216), HOXD10, Integrin, MAK, MAPK8, MBD1, MBD3 (includes EG:53615), NEDD9, NEUROD1, PAX6, PBX1, POU2F2, PRKAA1, PRKAA2, PRKCE, PTPN18, RREB1, RSBN1, SOX3, TOX4	Organismal Development, Cancer, Reproductive System Disease
3	41	32	ALP, BCL2L1, BMP2, BRPF1, CBX3, CCNE1, CGGBP1, CHRDL2, CXORF15, E2f, EIF3A, FMR1, GBX2, GOLM1, GRIK5, HIST1H1E, HIST1H3B (includes EG:8358), MYB, NOTCH1, PAX9, PMP22, POU4F2, PRR11, RAG2, RGS32, RORA, SHH, SKP2, SND1, Stat3-Stat3, TCF3, TCF21, TFE3, ZFAND3, ZP1	Cellular Growth and Proliferation, Cell Morphology, Hematological System Development and Function
4	41	32	ANAPC1, ARS2, ASXL2, BRD8, CLDND1, CROP, Cyclin B, ELL2, EZH2, GEMIN4, GTF3C1, Histone h3, ING3, KAT5, KIAA0265, KIAA1310, KPNA4, MED31, MNDA, MYBBP1A, NFE2, NOC2L, PBX3, PIN4, PNN, PRPF19, PRUNE, RBM33, RNA polymerase II, RRP1, SETMAR, TCEA1, TMPO, XIST, ZNF143	Gene Expression, DNA Replication, Recombination, and Repair, Cell-To-Cell Signaling and Interaction
5	38	31	AIFM2, ANKH, ARPC1B, C14ORF106, CADM1, CDC14B, CITED1, CNOT4, Creatine Kinase, GADD45, GIGYF2, GTSE1, Jnk dimer, NBR1, PRKAB2, PSRC1, PVRL1, PVRL3, RBBP6 (includes EG:5930), RRN3, SMA4, SNRK, SNRPN, STAG1, STK17A, TAF1A, TP53, UBA6, UBE2, UBE2D2, UBE2E1, UBE2E3, UBE3A, WAPAL, ZNF84	Developmental Disorder, Ophthalmic Disease, Cancer
6	36	30	ACY1, APAF1, BAG4, Caspase, CFLAR, CYCS (includes EG:54205), DEDD, DPPA2, DPPA4, FEM1B, Hsp70, Hsp90, HSPA6, HSPBP1, KHDC1, NLRP1, NLRP3, NOD1, NR3C2, OSBPL2, PDGF BB, PRPF4, RIPK2, RPS14, RYBP, SFRS1, SFRS7, SFRS10, Sphk, SRPK1, TNFAIP3, TRA2A, TRIOBP, U2AF2 (includes EG:11338), ZNF250	RNA Posttranscriptional Modification, Cell Death, Cancer
7	32	28	CR2, DLK1, ERK, F11R, Fgf, FGF4, FGF9, FGF10, Fgfr, FGFR1, FGFR2, FRS2, GPR132, IL17RD, LAMA3, Mmp, MMP7, MMP11, MMP28, NGEF, NODAL, NTRK2, Opsin, PCSK7, PLC gamma, RABGEF1, RAPGEF2, SAG, SERPINA1, SLC35E1, SLC9A3, SPZ1, Tgf beta, TGFA, ZNF384	Cellular Growth and Proliferation, Embryonic Development, Tissue Development
8	32	28	ARF6, ATF7IP, ATPase, BAT1, C2ORF13, CFD, Ck2, CSNK2B, CTSB, DDX19B, DHX8, FCGR1A/2A/3A, GGA1, KIAA1632, KRT13, KRT6B, MAX, MGA (includes EG:23269), MYH9, Myosin, Myosin Light Chain Kinase, NKX3-1, NPEPPS, PEX6, PIP5K1A, PTK2B, RAD51C, SENP3, SRPRB, STARD10, STXBP1, Tni, TRIM63, Tubulin, XPO7	Gene Expression, Molecular Transport, RNA Trafficking
9	30	27	ALDH7A1, ALS2CR2, AMH, BCL2L2, Bmpr1, BMPR1A, Caspase 3/7, CDH22, CHRNA3, CHRNA9, COMMD1 (includes EG:150684), CRIM2, FGG, HLA-F, IL1F6, Jnkk, Map3k, MAP3K2, MAP3K3, MEKK, MTSS1, NAIP, NF-κB, NFkB, Nicotinic acetylcholine receptor, OLR1, PLK3, PTPRD, SNIP1, ST8SIA1, TAP2, WNK1, XIAP, ZAK, ZNF33A	Amino Acid Metabolism, Posttranslational Modification, Small Molecule Biochemistry
10	30	27	BNIP3L, CAMTA1, CD3, CD8, CD247, CD8A, CLCA2 (includes EG:9635), CNOT1, CNOT2, CNOT6L, ERN1 (includes EG:2081), GAB3, GCN1L1, GRAP2, IL18R1, IL18RAP, LAT, LDB3, MAPK8IP2, MEF2, MHC Class I, MYH3, NFAT complex, NFATC1, P38 MAPK, PTPRC, RINT1, SCN5A, SLC9A8, SOS2, SYK/ZAP, TAOK2 (includes EG:9344), TCR, TRA@, TRIM27	Gene Expression, Hematological System Development and Function, Immune and Lymphatic System Development and Function

IPA score = −(log (P value)); IPA score is associated with the significance of the selected gene network. The higher the score, the more chance that it is true (from IPA).

**Table 2:** Top 10 biological function networks enriched in “reprogrammed transcriptome in R/B iSCNT 8- to 16-cell embryo.”

High level function	Significance	# Global analysis genes
Gene Expression	2.76E-10-5.49E-03	228
Cellular Growth and Proliferation	6.15E-10-5.17E-03	289
Cell Death	4.86E-09-5.49E-03	267
Cellular Development	9.17E-09-5.38E-03	233
Organism Development	5.77E-07-5.3E-03	101
Tissue Morphology	1.23E-06-5.49E-03	122
Organ Development	1.36E-06-4.39E-03	109
Nervous System Development and Function	1.67E-06-5.49E-03	104
Embryonic Development	1.81E-06-5.49E-03	120
Organ Morphology	2.04E-06-5.49E-03	30

Among the activated genes in the iSCNT embryo, the expression of 69 genes increased more than 50-fold, and a large portion of them — 14 genes — were transcription regulators, including POU2F2, ADNP, POLR3K, IRF6, FOXA2, HOXD10, JAZF1, and TUB.

### Validation of microarray data

**Figure 2:**
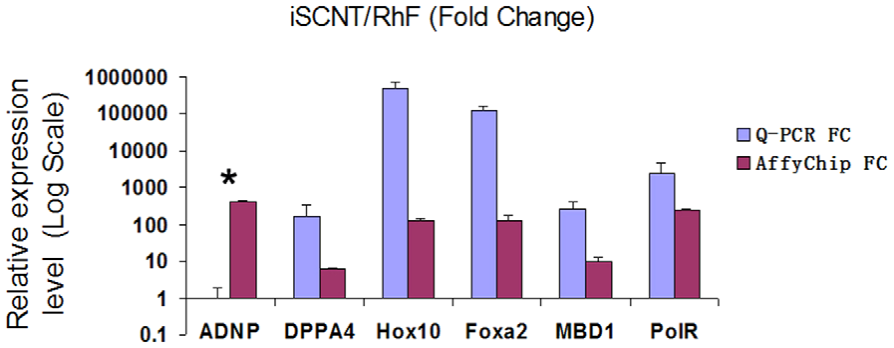
Quantitative RT-PCR verification of the gene chip array result. Ratio of gene expression between of iSCNT embryos vs. fibroblast cells by qRT-PCR (blue); and gene chip (red).

We used a list of selected genes (ADNP, DPPA4, Hox10, Foxa2, MBD1, and PolR3K) to validate the microarray result by qRT-PCR (Fig. 2). Table S7 lists the primers of these rhesus-specific genes. We used GAPDH house-keeping gene expression to normalize PCR results. Similar results of qRT-PCR and microarray showed up in most genes, except for ADNP (Fig. 2). These result verified reactivation of these genes during iSCNT.

### Common gene expression of iSCNT and rhesus IVF embryos and pluripotency of reprogrammed transcriptomes of iSCNT embryos

**Figure 3:**
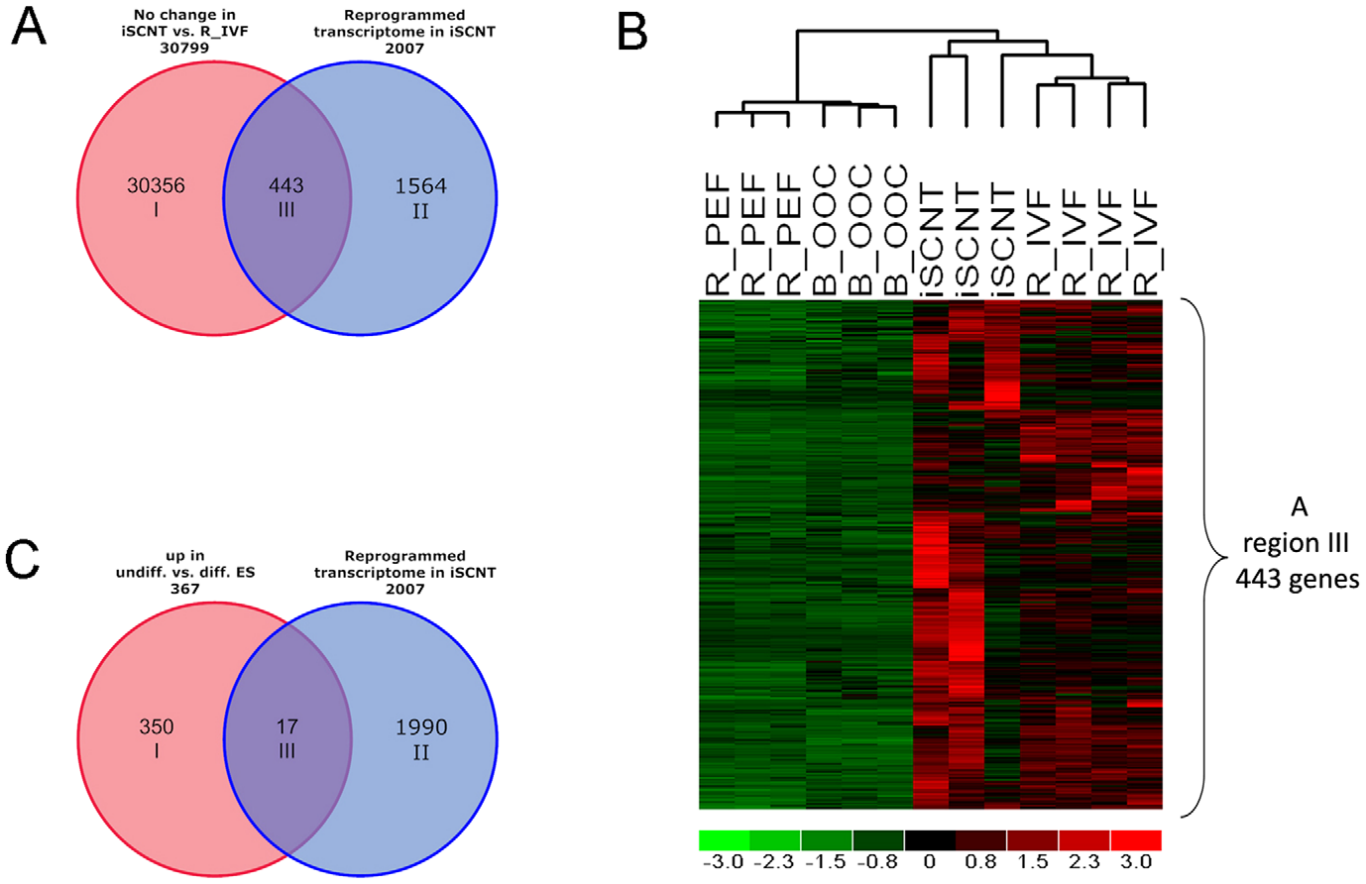
Characterizing the “reprogrammed transcriptome in R/B iSCNT 8- to 16-cell embryo.” **A.** Venn diagram showing common gene expression between iSCNT and rhesus IVF 8- to 16-cell embryo in region III (443 genes). Blue circle indicates reprogrammed transcriptome in R/B iSCNT 8- to 16-cell embryos (2,007 genes); red circle denotes transcripts that have similar expression levels between iSCNT embryos vs. rhesus IVF 8- to 16-cell embryos (R_IVF). **B.** Clustering analysis of region III, 443 genes. Red denotes high and green denotes low gene expression levels. **C.** Venn diagrams showing “the reprogrammed transcriptome in R/B iSCNT,” including 17 pluripotency-related genes in region III. Red circle indicates the number of upregulated genes in undifferentiated vs. differentiated rhesus ES (Rh_ES) [33]; blue circle denotes the ”reprogrammed transcriptome in R/B iSCNT 8- to 16-cell embryos (Fig. 3A region III).”

Microarray and qRT-PCR data showed evidence that reactivation of embryonic genes took place in R/B iSCNT. This left us wondering how accurate this reprogramming process was and whether the reprogrammed transcriptome of iSCNT was the same as the normal rhesus embryo. To answer these questions, we directly compared transcriptomes of iSCNT rhesus embryos with *in vitro* fertilized 8- to 16-cell rhesus embryos [8]. We found 30,356 transcripts with comparable expression levels shared between both sets of embryos. When we compared this list with the reprogrammed iSCNT transcriptome — 2,007 genes — we found 443 genes shared in both datasets (Fig. 3 A,B), indicating that the reprogrammed transcriptome in the iSCNT embryo partially resembled normal development or normal EGA gene expression in the IVF embryo. Tables S3 and S4 show the list of 443 genes and the functional analysis by IPA. In the context of SCNT, rebuilding pluripotency is an important step in the dedifferentiation process of a somatic cell [9]. We checked the pluripotency-related gene expression in the iSCNT embryo by comparing the reprogrammed transcriptome — 2,007 genes — with the rhesus ES cell upregulated gene list [10], and we found 17 pluripotency-related genes upregulated, including SALL4, DPPA2, and PRDM14 (Fig. 3C, Table S3). These reactivated ES-specific genes in iSCNT embryos indicate that the reprogrammed iSCNT transcriptome indeed assumes characteristics of pluripotency similar to the IVF embryo and rhesus ES cells.

### Incomplete reprogramming of rhesus genome in iSCNT

**Figure 4:**
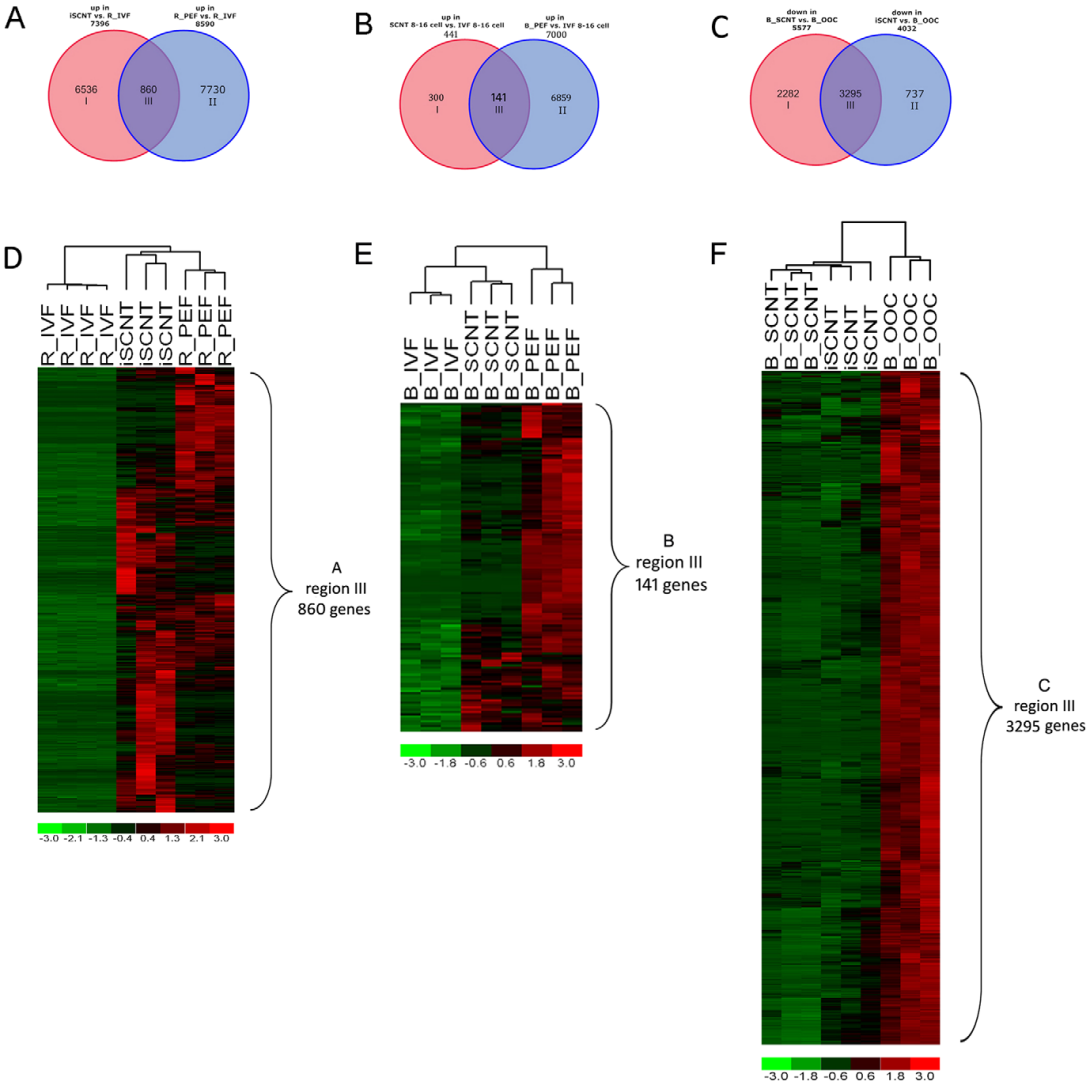
Incomplete reprogramming in R/B iSCNT and bovine SCNT embryo. **A.** Venn diagram showing somatic gene expression in 8- to 16-cell stage iSCNT embryos (region III, 860 genes) compared with IVF embryos. Red circle denotes upregulated genes in iSCNT compared to rhesus IVF 8- to 16-cell embryos (R_IVF), and blue circle denotes upregulated genes in rhesus fibroblast (R_PEF) compared to rhesus IVF 8- to 16-cell embryo (R_IVF). **B.** Venn diagram showing somatic gene expression in 8- to 16-cell stage SCNT embryos (region III, 141 genes) compared with bovine IVF embryos. Red circle denotes upregulated genes in SCNT compared to IVF embryos, and blue circle denotes upregulated genes in bovine fibroblast compared to IVF embryo. All samples were run on the bovine genome array. **C.** Venn diagram showing partial degradation of bovine maternal RNA in iSCNT embryo. Red circle denotes downregulated genes in the iSCNT embryo compared to the bovine oocyte (B_OOC); blue circle denotes downregulated genes in the bovine SCNT embryo compared to the bovine oocyte (B_OOC). All samples were run on the bovine genome array. **D.** Clustering analysis using leaky somatic genes found in iSCNT embryo (Fig. 4A region III, 860 genes). **E.** Clustering analysis using leaky somatic genes found in SCNT embryo (Fig. 4B region III, 141 genes). **F.** Clustering analysis by the common degraded maternal RNA in both SCNT/iSCNT embryos (Fig. 4C region III, 3295 genes). Red denotes high and green denotes low gene expression levels.

*In vivo* and *in vitro* development of SCNT embryos were reportedly lower than IVF embryo [11]. Incomplete genome reprogramming of the donor cell is believed to cause the failure of these embryos to thrive. In this study, we still observed abnormal fibroblast-specific gene expression in iSCNT embryos. We identified 860 genes showing high levels of expression in fibroblast donor cells and iSCNT embryos compared to rhesus IVF embryos (shown in Fig. 4A,D and listed in Table S5). Our results suggest that iSCNT embryos did not silence these genes completely. Among the most highly expressed somatic genes out of the 860 genes are Col1A1, Col3A1, and Col4A1, which are involved in the process of collagen production in fibroblasts. In SCNT embryos, we also found, to a lower extent, abnormal fibroblast-specific gene expression (Fig. 4B,E and listed in Table S6). These results suggest that neither iSCNT nor SCNT embryos can silence the donor-cell-specific genes — a phenomenon known as epigenetic memory — which likely contributes to developmental failures.

Before EGA, maternal RNA and protein play critical roles in reprogramming SCNT and iSCNT [12], [13]. As part of the maternal-to-zygote transition process, maternal RNA degradation takes place in parallel with ZGA and plays an important role in embryo development. Recent evidence has shown that newly transcripted TBP and Mir430 from the embryonic nuclei play important roles in maternal RNA degradation [14], [15], [16]. Therefore, we can infer that the proper degradation of maternal RNA could signal a successful EGA. By using the bovine genome array to compare bovine mRNA levels in iSCNT and same-species bovine SCNT array, our research found a significant drop of maternal RNA in both embryos (Fig. 4C,F). We compared the maternal degradation profiles between iSCNT and SCNT (5,577 versus 4,032 genes), and we found 3,295 genes shared in both sets of embryos. However, 2,282 genes were uniquely downregulated in the same-species SCNT embryos, much more than the 737 genes uniquely downregulated in iSCNT embryos (Fig. 4C). These results indicated that the SCNT embryo is capable of a more significant degradation of maternal RNA than the iSCNT embryo (Fig. 4). Thirteen well-characterized maternal transcripts [17] showed dynamic degradation in our study: BMP15, GDF9, ZP4, ZP3, ZP2, NFKBIA, EXT2, PPARG, SLBP, FRZB, c-MOS, STAT3, and DNMT1. We found that, in iSCNT embryos, ZP4, ZP3, ZP2, NFKBIA, EXT2, c-MOS, STAT3, and DNMT1 were degraded like they were in IVF embryos. However, BMP15, GDF9, PPARG, SLBP, and FRZB were not degraded as they were in bovine SCNT embryos. This result further implies that improper degradation of maternal transcripts may play a role in the proper development of iSCNT embryos.

## Discussion

iSCNT can be used to answer basic developmental biology questions, and as a tool to generate cells for regenerative medicine studies [5]. Successful iSCNT experiments [3], [18] prompted us to further investigate the mechanism of iSCNT reprogramming. Our prior iSCNT study, using an enucleated bovine oocyte and the genome of a chimpanzee somatic cell, demonstrated that EGA took place at the 8- to 16-cell stage [19]. However, we could not determine the extent of global genome reprogramming. In this study, we analyzed the transcriptome of an 8- to 16-cell rhesus-bovine iSCNT embryo at the time of EGA and found that, while no embryos were capable of developing beyond the morula stage, a large number of developmentally important rhesus-specific embryonic genes were reactivated by the bovine cytosol.

### Reprogramming and the EGA during iSCNT embryo development

During SCNT, the genome of the somatic cell must undergo vast epigenetic changes that will result in shutting down the donor's cell-type-specific RNA transcription pattern and begin to transcribe embryo-specific genes, ultimately regaining pluripotency. Others have already demonstrated that, in IVF embryos, improper EGA will cause death of the embryo [15]. SCNT embryos have the added challenge of activating the donor cell's genome in a fashion that resembles EGA concurrently with or subsequently to — the timing remains unclear — somatic-cell-specific gene silencing. The maternal RNA in the oocyte cytosol — including but not limited to gene transcripts related to pluripotency and chromatin remodeling [6] — is expected to play a key reprogramming in SCNT/iSCNT embryos. We speculated that monitoring the mRNA of the 8- to 16-cell iSCNT embryos would enable us to understand the extent to which these embryos begin to resemble a normally fertilized one.

In fertilized bovine embryos, the residue of maternal RNA remains high at the 8- to 16-cell stage [7]; therefore, the residual of maternal RNA must be subtracted from the embryo transcriptome in order to unveil embryo-specific transcripts present in the iSCNT embryos. In doing so, we found that 3,438 genes were differentially expressed. Further, we subtracted the genes expressed in the original rhesus fibroblasts, which left 2,007 differentially expressed in the iSCNT embryos. The top biological function of these reactivated genes was “gene expression,” indicating active transcriptional activity and strongly suggesting that EGA was taking place in the iSCNT embryos. This result accords with results reported in fertilized mice embryos at the 2- cell stage, where EGA transcriptome analysis determined that “gene expression” was the top biological function of the found upregulated genes [20]. About one quarter of the 2,007 reactivated genes that we found reached comparable levels of expression to the same-species IVF 8- to 16-cell rhesus embryos. This indicates that the reprogrammed genome recapitulates, to a certain extent, normal embryonic development. The proportion of reactivated genes — one quarter — remains low, suggesting incomplete reprogramming; however, the fact that we detected these genes after subtracting both the bovine oocyte and fibroblast transcriptomes from the iSCNT transcriptome indicates a degree of compatibility between the bovine cytosol and the rhesus genome. Our results differ from those recently reported by Chung et al. (2009) [21] ; they observed vastly different gene expression profiles at the 8- to 16-cell stage between same-species and iSCNT embryos. This could be explained by the fact that Chung et al. did not account for any residual maternal RNA from the oocyte cytoplasm that might have been present when they analyzed the iSCNT embryos.

### Incomplete reprogramming in iSCNT

Embryos cloned using SCNT procedures have exhibited more *in vivo* developmental defects than fertilized embryos. This phenomenon has been attributed to a “ripple effect” later in development from faulty epigenetic reprogramming early in development [22]. In general, whole-transcriptome analyses of SCNT embryos have failed to demonstrate a specific pattern in gene expression that points to a common origin of the reprogramming failures [3]. Instead, multiple studies in several different species have confirmed the stochastic nature of the developmental failures. These experimental shortcomings could be attributed to the fact that errors in genome reprogramming are likely subtle, remaining undetected when using current molecular tools. By using the oocyte from one species and the somatic cell of another, we expect to highlight some of these reprogramming errors, making them easier to detect. In this study, we found improper transcription of somatic genes and failures to reset or silence these genes after epigenetic reprogramming performed by the cytosol. The iSCNT embryo still expressed over 800 fibroblast-specific genes, suggesting that some somatic genes are more prone to incompletely reprogramming in the context of iSCNT. This finding was consistent with the research using mouse SCNT embryos [22], which found gene misexpression at the 2-cell stage of the mouse SCNT embryo (equivalent to the mouse EGA stage).

Others have shown that some remnants of maternal RNA can detrimentally affect embryonic development after EGA [23]. Reportedly, EGA and further embryo development require the degradation of maternal RNA [15]. That study found that c-mos, tPA and Gdf9 undergo rapid degradation after fertilization, suggesting the potentially detrimental effect of these maternal RNAs. In fact, when injected into 2-cell stage embryos, c-mos protein blocked embryo development beyond the 2-cell stage [24]. However, that analysis seldom extended to the degradation of maternal RNA in iSCNT embryos. Our study monitored the degradation of the maternal RNA global profile. We found broader maternal RNA degradation in SCNT embryos than in iSCNT embryos. Of interest, Gdf9 did not degrade in iSCNT embryos. As one of multiple reprogramming steps, faulty degradation of maternal RNA in iSCNT embryos could potentially be one of the causes of low reprogramming efficiency in iSCNT.

In summary, we determined the extent to which the genomes of 8- to 16-cell iSCNT embryos are reprogrammed, using same-species SCNT and IVF embryos as references. We analyzed the EGA-related transcriptional network and maternal RNA degradation. Our results showed that EGA occurred in iSCNT embryos and that the somatic genomes of the donor cells were partially reprogrammed. However, the epigenetic memory of the somatic cells remained greater in iSCNT embryos, suggesting a deficiency in species-specific gene silencing, perhaps through microRNA processing that has not yet been described. Further functional studies of gain and loss of function using the genes unveiled here may help us to optimize not only the SCNT but the iSCNT technique, as well.

## Materials and Methods

### Embryo production

We collected recipient bovine oocytes and matured them *in vitro* using procedures previously described [19]. Briefly, we obtained bovine oocytes by aspirating follicles on slaughterhouse-derived ovaries. We cultured immature cumulus-oocyte complexes in Tissue Culture Medium 199 (TCM-199) (Sigma, St Louis, MO) supplemented with 10% fetal calf serum (FCS), 0.2 mM pyruvate, 25 µl/ml gentamicin, 0.5 µg/ml Luteinizing Hormone (LH; Sioux Biochemical, Sioux Center, IA) and 1 µg/ml estradiol-17β for 16 to 18 hours at 38.5°C with 5% CO_2_ in the air. Eighteen hours after the start of maturation, cumulus cells were removed from the oocytes, and oocytes with extruded first polar bodies were selected as MII oocyte and used for enucleation. We labeled oocytes with DNA fluorochrome (Hoechst 33342) (Sigma, St Louis, MO) before enucleation. The MII plate was removed by aspiration, using an enucleation pipette; to ensure removal of the oocyte chromatin, we exposed the aspirated cytoplasm to UV light to examine of the outcome of the enucleation. We used only those oocytes that were successfully enucleated.

We used adult rhesus fibroblast cells, kindly provided by Dr S. Mitalipov (Oregon National Primate Research Center), as donor cells for rhesus/bovine nuclear transfer, successfully establishing embryonic stem (ES) cells [10]. Bovine adult fibroblasts were used as donor cells for bovine/bovine nuclear transfer experiments. Fibroblast cells were cultured in Dulbecco's Modified Minimum Essential Medium (DMEM; Gibco BRL, Grand Island, NY) supplemented with 10% FCS (Hyclone, Logan, UT) at 37°C under a gas phase of 5% CO_2_ in air at high humidity until they reached approximately 70% confluence. Before nuclear transfer, single cells were obtained by pronase treatment (100 µg/ml).

For the production of SCNT embryos we used a micropipette to transfer a single donor cell into the perivitelline space of each enucleated oocyte. Then, we fused nuclear transfer couplets in sorbitol fusion medium by applying a single electric pulse (20 µs pulse, 2.4 kV/cm). One hour after fusion, the fused embryos were selected. We activated SCNT/iSCNT embryos using 5 µM of ionomycin (Calbiochem, La Jolla, CA) for four minutes, followed by four hours of treatment with 5 µg/ml 6-DMAP (Sigma, St Louis, MO). At the end of incubation, activated SCNT/iSCNT embryos were cultured in KSOM medium (50 embryos/100 µl drop) for 64 to 65 hours to reach the 8- to 16-cell stage after the embryos were activated by 6-DMAP.

In vitro fertilized Rhesus embryos were produced as previously described [8]. Briefly, cumulus-oocyte complexes (COCs) were collected by ultrasound-guided aspiration from female rhesus monkey and then cultured in TL-PVA medium. In vitro fertilization was performed by standard IVF procedures of rhesus macaque oocytes. Sperm were washed from seminal plasma and resuspended in TL-BSA medium. After adding sperm, the next morning, oocytes were transferred into chemically defined, protein-free hamster embryo culture medium 9 (HECM-9) incubated at 37°C in a humidified atmosphere of 5% CO_2_, 10% O_2_, and 85% N_2_.

### Embryo collection and RNA isolation and gene expression analysis

After checking their nuclei by labeling with 0.5 µg/ml of Hoechst 33342 for 20 minutes, we selected groups of ten 8- to 16-cell iSCNT/SCNT embryos and bovine MII oocytes 64 to 65 hours after activation, rinsed them in sterile PBS, and lysed them in 20 µl of extraction buffer (Arcturus, Mountain View, CA). Each sample was incubated for 30 minutes at 42°C, centrifuged at 3000×g for 2 minutes, and stored at −80°C until use. We isolated total RNA using the PicoPure RNA Isolation Kit (Arcturus), following the manufacturer's instructions. All RNA samples within the purification column were treated with RNase-Free DNase (Qiagen, Valencia, CA) and eluted with RNase-free elution buffer. Extracted RNA was stored at −80°C until use. We collected fibroblast cells and isolated total RNA using the same kit.

We performed reverse transcription (RT) and complementary DNA (cDNA) amplication based on a previously published protocol [6] [19]. Briefly, we mixed 5 µl total RNA with 300 ng of anchored T7-Oligo(dT)_24_V Promoter Primer (Ambion, Austin, TX). After denaturation, we added the following reagents to each reaction tube: 1.4 µl of SMART II A oligonucleotide (5′AAGCAGTGGTATCAACGCAGAGTACGCGrGrGr-3′) (Clontech, Mountain View, CA), 4 µl of 5× first-strand buffer, 2 µl of 20 mM DTT, 0.6 µl of 5 µg/µl T4 Gene 32 Protein (Roche, Indianapolis, IN), 2 µl of 10 mM dNTPs, 20 U of RNase Inhibitor (Ambion), and 1 µl of PowerScript Reverse Transcriptase (Clontech). Total reaction volume was 20 µl. After gentle mixing, the reaction tubes were incubated at 42°C for 60 minutes in a hot-lid thermal cycler. We terminated the reaction and purified the cDNA using the NucleoSpin Extraction Kit (Clontech), following the manufacturer's instructions. We amplified the cDNA by PCR reaction, based on Advantage 2 Taq (Clonetech) and a primer pair: 5′SMART upper primer (5′-AAGCAGTGGTATCAACGCAGAGTA-3′), 3′SMART lower primer (5′-CGGTAATACGACTCACTATAGGGAGAA-3′). PCR was performed in the following conditions: 95°C for 1 minute followed by 19 cycles, each consisting of denaturation at 94°C for 30 seconds, annealing at 62°C for 30 seconds, and extension at 68°C for 10 minutes. Again, we purified using a NucleoSpin Extraction Kit (Clontech).

The purified cDNA was IVTed and biotin-labeled using the BioArray HighYield RNA Transcript Labeling Kit with T7 RNA polymerase (Enzo, Farmingdale, NY) as described by the manufacturer. The biotin-labeled aRNA was purified using RNeasy mini columns (RNeasy Mini Kit, Qiagen). We fragmented 15 µg of the labeled aRNA at 94°C for 35 minutes in a 1× fragmentation buffer (40 mM Tris–acetate, pH 8.1, 100 mM KOAc, 30 mM MgOAc). We used the Affymetrix GeneChip System (Affymetrix, Santa Clara, CA) to hybridize, stain, and image the arrays, using published protocols [6] [25].

We analyzed the scanned array images using dChip [26], which is more robust than the Affymetrix Software Microarray Analysis Suite 5.0 in signal calculation for about 60% of genes [27]. In the dChip analysis, a smoothing spline normalization method was applied prior to obtaining model-based gene expression indices, a.k.a. signal values. The dChip analysis identified no outliers, so all samples were carried on for subsequent analysis.

When comparing two groups of samples to identify genes enriched in a given group, we used the lower confidence bound (LCB) of the fold change (FC) between the two groups as the cut-off criterion. If 90% LCB of FC between the two groups was above 1.2, we considered the corresponding gene to be differentially expressed. The LCB stringently estimates the FC and has proved to be the better ranking statistic [26]. The dChip LCB method for assessing differentially expressed genes has proved superior to other commonly used approaches [28], such as MAS 5.0 and methods based on the Robust Multiarray Average [29]. By using the LCB, we can be 90% confident that the actual FC is some value above the reported LCB. Using custom arrays and quantitative reverse transcriptase real-time PCR (qRT-PCR), research has suggested that Affymetrix chips may underestimate differences in gene expression [30]; a criterion of selecting genes that have an LCB above 1.2 most likely corresponds to genes with “actual” fold changes of at least 3 in gene expression [31].

After having calculated gene lists that are differentially expressed between various groups, we identified genes following certain patterns — such as genes that are iSCNT specific with comparable levels of expression in IVF samples — through set intersection. We used the Ingenuity Software Knowledge Base (IKB; Redwood City, CA) to analyze differentially expressed gene lists and genes with certain patterns of expression. To this end, we identified functional categories and known biological pathways in input to the IKB that are represented beyond random chance using hypergeometric distribution and multiple hypothesis testing (p<0.05). We performed hierarchical clustering of samples and genes using average linkage clustering with Euclidean distance as the metric of similarity to construct an “unweighted pair group method with arithmetic mean” (UPGMA) tree [32]. We normalized the signal values used for clustering to expression levels that have been subject to row normalization. All data is MIAME compliant and that the raw data has been deposited in GEO (accession number in process).

Validation of the microarray findings was done using qRT-PCR. We performed PCR reactions in an Applied Biosystem 7000 real-time machine as follows: 50 for 2 minutes, 95 for 10 minutes, and 40 cycles of 95 for 15 seconds and 60 for 1 minute. A dissociation curve for product specificity test was run at the end of the PCR reaction. We performed three biological replicates and used duplicate samples for each biological replicate. We normalized the result using a house-keeping gene (rhesus GAPDH).

## Supporting Information

Figure S1 **The DNA array sample list used in this research.** A. Clustering diagram of transcriptomes of rhesus fibroblast (R_PEF), rhesus IVF 8- to 16-cell embryos (R_IVF), bovine fibroblast (B_PEF), 8- to 16-cell stage iSCNT embryos (iSCNT), and bovine oocyte (B_OOC) in rhesus genome array by genes called present in these arrays. B. Clustering diagram of transcriptomes of rhesus fibroblast (R_PEF), bovine fibroblast (B_PEF), bovine 8- to 16-cell stage SCNT embryos (B_SCNT), bovine IVF 8- to 16-cell embryos (B_IVF), bovine oocyte (B_OOC), and 8- to 16-cell stage iSCNT embryos (iSCNT) in rhesus genome array by genes called present in these arrays.
(TIF)

Table S1 **Correlation between samples in rhesus and bovine genome array.**
(XLS)

Table S2 **“Reprogrammed iSCNT transcriptome” of 2007 genes.**
(XLS)

Table S3 **Common gene expression of iSCNT and rhesus IVF embryo of 443 genes.**
(XLS)

Table S4 **Top 10 function of common gene expression of iSCNT and rhesus IVF embryo of 443.**
(XLS)

Table S5 **Leaking expression of somatic specific genes in R/B iSCNT embryo.**
(XLS)

Table S6 **Leaking expression of somatic specific genes in bovine SCNT embryo.**
(XLS)

Table S7 **Primer list for validating microarray data by qRT-PCR.**
(XLS)

## References

[1] Memili E, First NL (1998). Developmental changes in RNA polymerase II in bovine oocytes, early embryos, and effect of alpha-amanitin on embryo development.. Mol Reprod Dev.

[2] Schultz RM (1993). Regulation of zygotic gene activation in the mouse.. Bioessays.

[3] Beyhan Z, Iager AE, Cibelli JB (2007). Interspecies nuclear transfer: implications for embryonic stem cell biology.. Cell Stem Cell.

[4] Tecirlioglu RT, Guo J, Trounson AO (2006). Interspecies somatic cell nuclear transfer and preliminary data for horse-cow/mouse iSCNT.. Stem Cell Rev.

[5] Dominko T, Mitalipova M, Haley B, Beyhan Z, Memili E (1999). Bovine oocyte cytoplasm supports development of embryos produced by nuclear transfer of somatic cell nuclei from various mammalian species.. Biol Reprod.

[6] Kocabas AM, Crosby J, Ross PJ, Otu HH, Beyhan Z (2006). The transcriptome of human oocytes.. Proc Natl Acad Sci U S A.

[7] Misirlioglu M, Page GP, Sagirkaya H, Kaya A, Parrish JJ (2006). Dynamics of global transcriptome in bovine matured oocytes and preimplantation embryos.. Proc Natl Acad Sci U S A.

[8] VandeVoort CA, Mtango NR, Lee YS, Smith GW, Latham KE (2009). Differential effects of follistatin on nonhuman primate oocyte maturation and pre-implantation embryo development in vitro.. Biol Reprod.

[9] Alberio R, Campbell KH, Johnson AD (2006). Reprogramming somatic cells into stem cells.. Reproduction.

[10] Byrne JA, Pedersen DA, Clepper LL, Nelson M, Sanger WG (2007). Producing primate embryonic stem cells by somatic cell nuclear transfer.. Nature.

[11] Cibelli J (2007). Development. Is therapeutic cloning dead?. Science.

[12] Kikyo N, Wolffe AP (2000). Reprogramming nuclei: insights from cloning, nuclear transfer and heterokaryons.. J Cell Sci.

[13] Bultman SJ, Gebuhr TC, Pan H, Svoboda P, Schultz RM (2006). Maternal BRG1 regulates zygotic genome activation in the mouse.. Genes Dev.

[14] Giraldez AJ, Mishima Y, Rihel J, Grocock RJ, Van Dongen S (2006). Zebrafish MiR-430 promotes deadenylation and clearance of maternal mRNAs.. Science.

[15] Alizadeh Z, Kageyama S, Aoki F (2005). Degradation of maternal mRNA in mouse embryos: selective degradation of specific mRNAs after fertilization.. Mol Reprod Dev.

[16] Ferg M, Sanges R, Gehrig J, Kiss J, Bauer M (2007). The TATA-binding protein regulates maternal mRNA degradation and differential zygotic transcription in zebrafish.. Embo J.

[17] Kues WA, Sudheer S, Herrmann D, Carnwath JW, Havlicek V (2008). Genome-wide expression profiling reveals distinct clusters of transcriptional regulation during bovine preimplantation development in vivo.. Proc Natl Acad Sci U S A.

[18] Chen Y, He ZX, Liu A, Wang K, Mao WW (2003). Embryonic stem cells generated by nuclear transfer of human somatic nuclei into rabbit oocytes.. Cell Res.

[19] Wang K, Beyhan Z, Rodriguez RM, Ross PJ, Lager AE (2009). Bovine Ooplasm Partially Remodels Primate Somatic Nuclei following Somatic Cell Nuclear Transfer.. Cloning Stem Cells.

[20] Zeng F, Schultz RM (2005). RNA transcript profiling during zygotic gene activation in the preimplantation mouse embryo.. Dev Biol.

[21] Chung Y, Bishop CE, Treff NR, Walker SJ, Sandler VM (2009). Reprogramming of human somatic cells using human and animal oocytes.. Cloning Stem Cells.

[22] Vassena R, Han Z, Gao S, Baldwin DA, Schultz RM (2007). Tough beginnings: alterations in the transcriptome of cloned embryos during the first two cell cycles.. Dev Biol.

[23] Paynton BV, Rempel R, Bachvarova R (1988). Changes in state of adenylation and time course of degradation of maternal mRNAs during oocyte maturation and early embryonic development in the mouse.. Dev Biol.

[24] Sagata N, Watanabe N, Vande Woude GF, Ikawa Y (1989). The c-mos proto-oncogene product is a cytostatic factor responsible for meiotic arrest in vertebrate eggs.. Nature.

[25] Beyhan Z, Ross PJ, Iager AE, Kocabas AM, Cunniff K (2007). Transcriptional reprogramming of somatic cell nuclei during preimplantation development of cloned bovine embryos.. Dev Biol.

[26] Li C, Wong WH (2001). Model-based analysis of oligonucleotide arrays: expression index computation and outlier detection.. Proc Natl Acad Sci U S A.

[27] Barash Y, Dehan E, Krupsky M, Franklin W, Geraci M (2004). Comparative analysis of algorithms for signal quantitation from oligonucleotide microarrays.. Bioinformatics.

[28] Shedden K, Chen W, Kuick R, Ghosh D, Macdonald J (2005). Comparison of seven methods for producing Affymetrix expression scores based on False Discovery Rates in disease profiling data.. BMC Bioinformatics.

[29] Irizarry RA, Bolstad BM, Collin F, Cope LM, Hobbs B (2003). Summaries of Affymetrix GeneChip probe level data.. Nucleic Acids Res.

[30] Yuen T, Wurmbach E, Pfeffer RL, Ebersole BJ, Sealfon SC (2002). Accuracy and calibration of commercial oligonucleotide and custom cDNA microarrays.. Nucleic Acids Res.

[31] Ramalho-Santos M, Yoon S, Matsuzaki Y, Mulligan RC, Melton DA (2002). “Stemness”: transcriptional profiling of embryonic and adult stem cells.. Science.

[32] Soumare S, Losfeld J, Blondeau R (1973). [Numerical taxonomy in the study of bacterial soil microflora in the north of France].. Ann Microbiol (Paris).

[33] Byrne JA, Mitalipov SM, Clepper L, Wolf DP (2006). Transcriptional profiling of rhesus monkey embryonic stem cells.. Biol Reprod.

