# Correction: Reprogrammed Transcriptome in Rhesus-Bovine Interspecies Somatic Cell Nuclear Transfer Embryos

**DOI:** 10.1371/annotation/5aa90741-1a8a-4443-83b1-c5abcb59bb51

**Published:** 2012-10-11

**Authors:** Kai Wang, Hasan H. Otu, Ying Chen, Young Lee, Keith Latham, Jose B. Cibelli

The following information was erroneously omitted from the published Funding statement:

"In addition, this research was supported by grants from the National Institutes of Health, and The Office of Research Infrastructure Programs (ORIP) Division of Comparative Medicine, and the National Center for Research Resources (NCRR), R24 OD-012221/R24RR015253 (KEL)." 

