# Supplementary figures and images for: Reprogrammed Transcriptome in Rhesus-Bovine Interspecies Somatic Cell Nuclear Transfer Embryos
Source: PLoS One. 2011 Jul 25;6(7):e22197. doi: 10.1371/journal.pone.0022197 (PMC3143123; doi:10.1371/journal.pone.0022197)

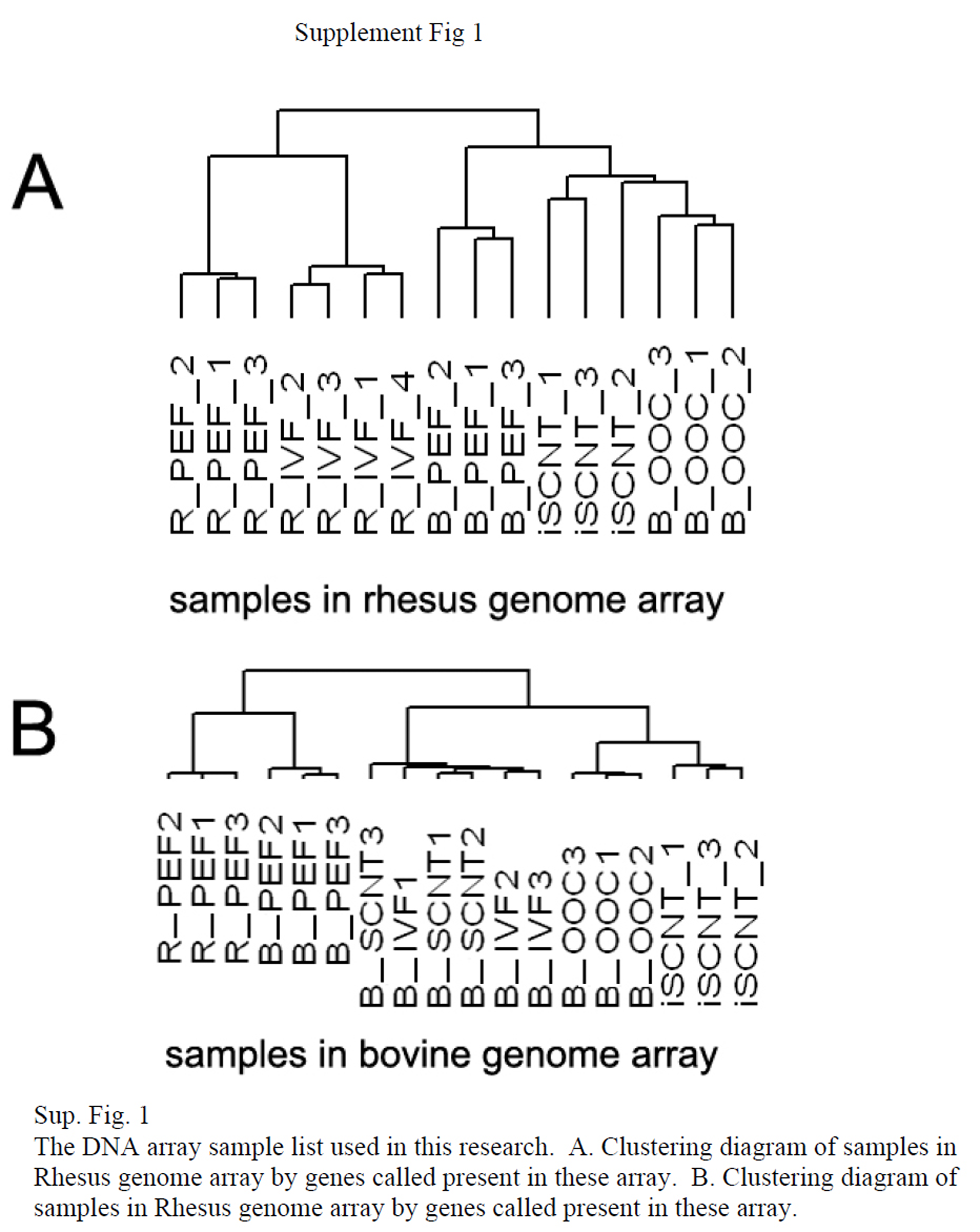

Supplement: Figure S1 — The DNA array sample list used in this research. A. Clustering diagram of transcriptomes of rhesus fibroblast (R_PEF), rhesus IVF 8- to 16-cell embryos (R_IVF), bovine fibroblast (B_PEF), 8- to 16-cell stage iSCNT embryos (iSCNT), and bovine oocyte (B_OOC) in rhesus genome array by genes called present in these arrays. B. Clustering diagram of transcriptomes of rhesus fibroblast (R_PEF), bovine fibroblast (B_PEF), bovine 8- to 16-cell stage SCNT embryos (B_SCNT), bovine IVF 8- to 16-cell embryos (B_IVF), bovine oocyte (B_OOC), and 8- to 16-cell stage iSCNT embryos (iSCNT) in rhesus genome array by genes called present in these arrays. (TIF) [file pone.0022197.s001.tif]
